# Incidental synchronous intrathyroidal parathyroid carcinomas and papillary thyroid microcarcinoma with compressive neck mass and primary hyperparathyroidism: case report and literature review

**DOI:** 10.1186/s12902-024-01656-8

**Published:** 2024-07-25

**Authors:** Tianfeng Xu, Xun Zheng, Tao Wei

**Affiliations:** Division of Thyroid Surgery, Department of General Surgery, West China Hospital, Sichuan University, No. 37 Guoxue Lane, Wuhou District, Chengdu, Sichuan Province China

**Keywords:** Parathyroid carcinoma, Intrathyroidal parathyroid, Papillary thyroid carcinoma, Hyperparathyroidism

## Abstract

**Background:**

Parathyroid carcinoma (PC) is a rare malignancy, often diagnosed incidentally through postoperative pathological examination. The occurrence of nodular goiter, intrathyroidal parathyroid carcinoma, contralateral parathyroid adenoma (PA), and papillary thyroid microcarcinoma (PTMC) is extremely uncommon, which prompted us to report our case experience.

**Case presentation:**

We describe a 67-year-old male who presented with a cervical mass causing tracheal compression, which prompted him to seek medical advice. Based on preoperative auxiliary examination results from color Doppler ultrasound, SPECT parathyroid imaging, and blood tests, he was initially diagnosed with a suspected parathyroid adenoma and nodular goiter. Excision of the right lobe and isthmus of the thyroid, and left superior parathyroid gland was conducted, which were sent to intraoperative frozen pathological examination. During intraoperative observation, adhesion around the right thyroid lobe was discovered. Consequently, right central area lymph node dissection was performed due to suspicion of an aggressive malignant tumor. Histology and immunohistochemistry analysis revealed incidental intrathyroidal parathyroid carcinoma, contralateral parathyroid adenoma, classical papillary thyroid microcarcinoma, and nodular goiter.

**Conclusion:**

Parathyroid carcinoma should be highly suspected when extremely high levels of PTH and severe hypercalcemia are present, which cannot be simply explained by a preoperatively localized parathyroid adenoma, especially when suspicious malignant adhesion is found during intraoperative exploration. In cases where multifocal thyroid nodules are associated with increased uptake of 99Tc-sestamibi, the possibility of coexisting carcinomas should be considered, not only for thyroid malignancy but also for the potential presence of intrathyroidal parathyroid carcinoma.

## Introduction

Parathyroid carcinoma (PC) is a rare endocrine neoplasm, accounting for only 0.005% of all malignancies. It is an uncommon cause of sporadic primary hyperparathyroidism, with the simultaneous occurrence probability being less than 1% [1]. Ectopic parathyroid glands, resulting from aberrant migration during embryogenesis, have an incidence of approximately 16% [2]. The coexistence of synchronous intrathyroidal parathyroid carcinoma, hyperparathyroidism, thyroid carcinoma, and nodular goiter is even rarer.

The management of PC presents challenges in clinical practice, as the diagnosis is mainly established through postoperative histological examination, and clinical manifestations and investigation results often lean towards a diagnosis of parathyroid adenoma. Moreover, intrathyroidal parathyroid carcinoma increases the difficulty of lesion localization. In this report, we describe a case of synchronous multiple intrathyroidal parathyroid carcinoma and provide a literature review to emphasize the diagnostic and treatment challenges from both endocrine and oncology perspectives. The patient provided written informed consent for the publication of this case report.

## Case presentation

A 63-year-old male presented with bilateral neck masses, accompanied by xerostomia, polyuria, constipation, and emotional irritability. He was initially diagnosed with hypercalcemia (serum calcium:3.75mmol/L), hyperparathyroidism (parathyroid hormone < PTH>:739pg/ml = 81.29pmol/L), and thyroid mass at a local hospital, where he received fluid infusion, diuresis, and calcitonin for symptomatic treatment. He then sought further treatment at the emergency department of our medical center. Upon admission, laboratory results revealed normal thyroid function but elevated serum calcium levels of 3.10 mmol/L (normal range 2.11–2.52), phosphate levels of 0.55 mmol/L (0.85–1.51), and PTH levels of 67.4 pmol/L (1.60–6.90). The patient experienced anorexia and weight loss since the onset of his illness. His previous medical history, which included kidney stones and bone fractures caused by trauma, drew our attention. However, no kidney disorders were detected, with serum creatinine levels of 90 μmol/L (68–108). There was no known family history of parathyroid disease, thyroid carcinoma, or endocrine neoplasia.

**Fig. 1:**
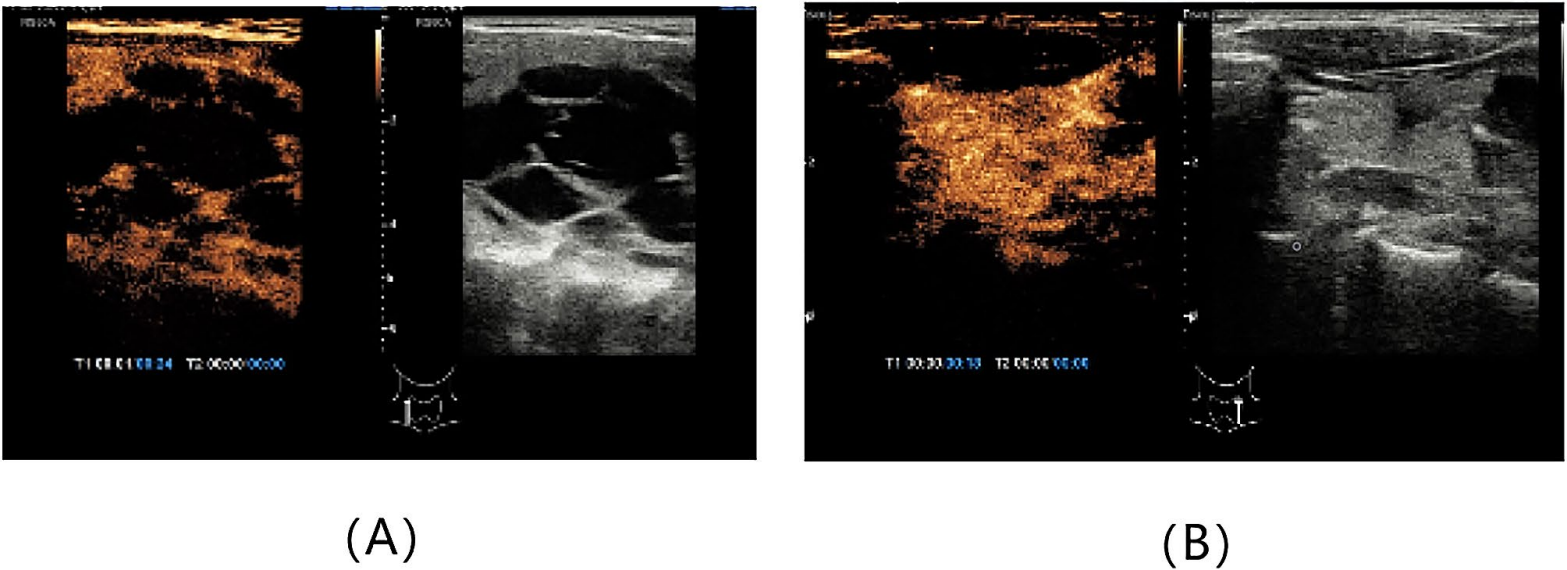
Thyroid contrast-enhanced ultrasound (CE-US) images. Multiple nodular goiter (**A**) and a parathyroid adenoma located in the middle and lower left thyroid lobe (**B**)

**Fig. 2:**
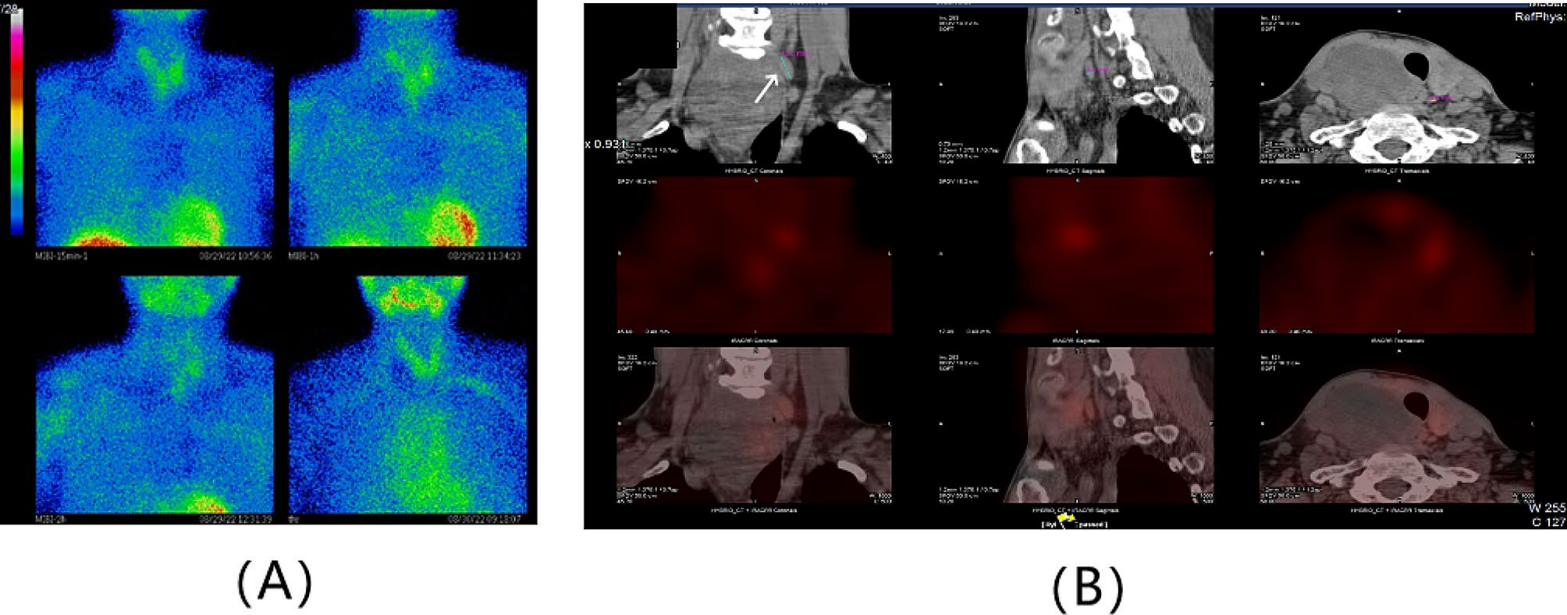
Slight uptake signal at the posterior tubercle of the superior left thyroid lobe in 99Tc-sestamibi dual-phase SPECT (**A**) and parathyroid fusion imaging showed parathyroid adenoma in 15.5 mm*4.3 mm*4.8 mm (**B**)

**Fig. 3:**
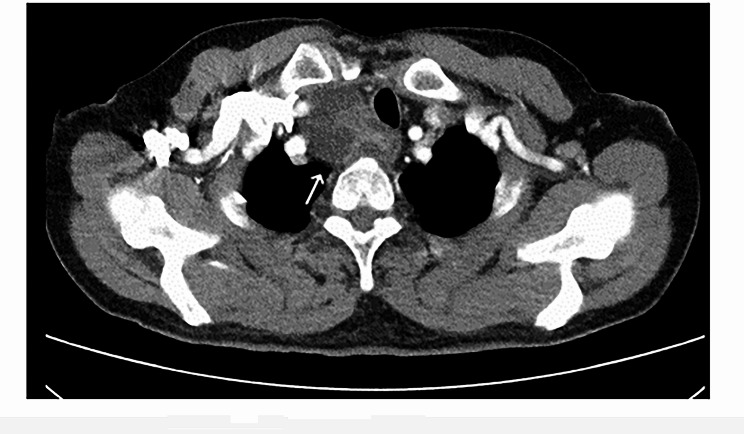
CE-CT demonstrated compression and displacement of the trachea and esophagus to the left by the nodule in the right thyroid lobe

Physical examination revealed a painless, approximately three-centimeter diameter nodule in the right thyroid lobe. Subsequently, imaging examinations were performed to localize and characterize suspicious lesions. Thyroid contrast-enhanced ultrasound (CE-US) suggested the presence of multiple nodular goiter and a parathyroid adenoma located in the middle and lower left thyroid lobe (Fig. 1). 99Tc-sestamibi dual-phase single-photon emission computed tomography (SPECT) revealed a slight uptake signal at the posterior tubercle of the superior left thyroid lobe, with no signals detected in other sites, which appeared to contradict ultrasound findings (Fig. 2). Neck contrast-enhanced computed tomography (CE-CT) demonstrated compression and displacement of the trachea and esophagus to the left by the nodule in the right thyroid lobe (Fig. 3).

**Fig. 4:**
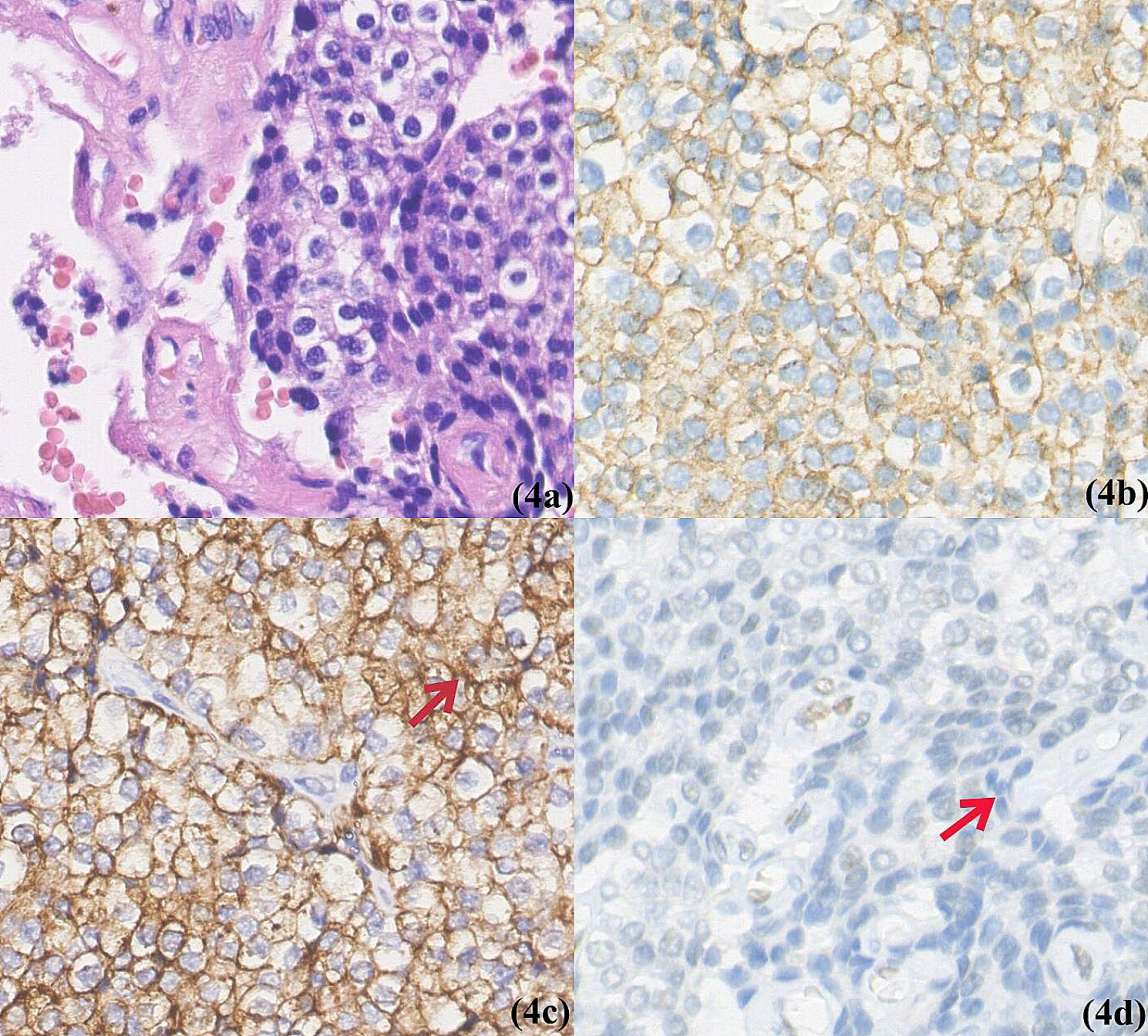
Histology and Immunohistochemical of intrathyroidal parathyroid carcinoma. (**4a**) The tumor is infiltrative within the thyroid. The expressions of chromogranin A (**4b**), PTH (**4c**), and Rb (**4d**) indicate that the cells originated in the parathyroid gland

The preoperative diagnosis included primary hyperparathyroidism with suspected left superior parathyroid adenoma, hypercalcemia, right thyroid lobe mass with tracheal compression, and nodular goiter. The initial surgical plan involved excision of the right thyroid lobe and isthmus, as well as left superior parathyroidectomy. The left superior parathyroid was confirmed as a parathyroid adenoma (PA) through intraoperative frozen section analysis. The left inferior parathyroid was normal parathyroid, for a portion of the left inferior parathyroid tissue was sent for intraoperative frozen examination. And we noticed the right superior parathyroid gland in the usual position, but after careful exploration (including within the thymus), no right lower parathyroid gland was found. During neck exploration, adhesions surrounding the right thyroid lobe raised suspicions of malignancy, prompting a right central area lymph node dissection. Multiple nodules were identified in the right thyroid lobe and isthmus; histological examination revealed that both a 2 cm x 2 cm x 1 cm grayish-white solid nodule and a 3 cm x 2 cm x 1 cm cystic nodule as parathyroid carcinoma (PC), with capsular and vascular invasion. Immunohistochemical staining demonstrated positivity for PTH and chromogranin A(CgA) and negativity for thyroid transcription factor-1(TTF-1), thyroglobulin(TG), retinoblastoma gene (Rb) and calcitonin (Fig. 4). The Ki-67 index was 3%. An additional grayish-white nodule measuring 0.4 cm x 0.3 cm x 0.3 cm adjacent to the capsule was identified as incidental classical papillary thyroid carcinoma, while other visible multiple cystic nodules were determined to be nodular goiter, the largest of which measured 1.5 cm x 1.5 cm x 1.2 cm. No cancer metastasis was found in the two lymph nodes dissected from the right central area.

No severe postoperative complications were observed. Serum calcium levels significantly decreased to 2.29 mmol/L, and PTH levels were reduced to 1.10 pmol/L on the first day after surgery. The patient received appropriate calcium supplementation, resulting in serum calcium levels of 1.90 mmol/L and PTH levels of 1.50 pmol/L at discharge on the third postoperative day. The patient continued oral calcium and calcitriol therapy after hospital discharge. One month after surgery, serum calcium and PTH levels were 1.96 mmol/L and 20.66 pmol/L, respectively, and 2.43 mmol/L and 11.12 pmol/L at three months postoperatively. The most recent measurements, taken six months after surgery, revealed serum calcium levels of 2.35 mmol/L and PTH levels of 8.03 pmol/L. Thyroid function remained normal throughout the perioperative and follow-up periods. No tumor recurrence or postoperative complications occurred during the six-month follow-up period.

## Discussion

In our case, four distinct pathologies were identified, including two intrathyroidal parathyroid carcinomas, papillary thyroid microcarcinoma, contralateral parathyroid adenoma, and nodular goiter. We conducted a literature review of the past 20 years, focusing on synchronous intrathyroidal parathyroid carcinomas and incidental papillary thyroid microcarcinoma (PTMC) or parathyroid adenoma (PA), aiming to elucidate the unique relationship and provide management recommendations based on these findings.

### Incidental PTMC

The prognosis of papillary thyroid carcinoma (PTC) is excellent, with a 5-year mortality rate of less than 2% [3]. PTMC is characterized as a papillary thyroid carcinoma with a diameter ≤ 1 cm. In recent years, the incidence of PTMC has increased, particularly among patients who undergo surgery for benign thyroid disease. Nodular goiter is the most common benign thyroid disease associated with PTMC [4], which is also observed in our case. Studies have reported that incidentally diagnosed PTMC is less invasive than clinically diagnosed PTMC preoperatively; however, their prognoses are essentially equivalent, and neither impacts the overall survival rate [5, 6].

### Intrathyroidal parathyroid tumor

Ectopic parathyroid glands result from aberrant migration during early development, with a prevalence of approximately 2–43%. Inferior parathyroids are more frequently found within the thyroid compared to superior parathyroids [7]. The incidence of intrathyroidal PAs is rare, ranging from 1 to 6%, while the rate of intrathyroidal PC may be even lower, considering the rarity of PC itself (see above). Both conditions present diagnostic challenges that can complicate treatment measures [8].

Initial clinical manifestations of patients with PTC combined with primary hyperparathyroidism (PHPT) can vary, with some being nonspecific, such as asymptomatic neck mass, hypercalcemia with or without symptoms, or fatigue [9], or even normal total serum calcium and PTH levels [10]. Others may experience kidney damage and bone pain [11]. Ultrasonography (US) is typically an effective and economical method for diagnosing thyroid and parathyroid diseases. A previously reported patient presented with fatigue and hyperparathyroidism; US revealed a large, lobulated, solid intrathyroidal nodule consisting of hypoechoic components with microcalcifications and hyperechoic components with vascularity, ultimately confirmed as PTC and ectopic parathyroid adenoma [12].

The concurrent presence of parathyroid adenoma and parathyroid carcinoma is exceedingly rare, and no unique diagnostic criteria exist for reference. A patient with modest hypercalcemia was ultimately diagnosed with both PA and PC, despite remarkably elevated parathormone levels, several times the normal range [13]. Synchronous PC and PA should be considered when a parathyroid lesion is abnormally enlarged and densely adherent to the thyroid lobe, particularly if there is no significant decrease in intraoperative PTH levels following the specific diagnosis and resection of PA [14]. During meticulous exploration of the excised thyroid lobe, normal ectopic parathyroid tissue should be transplanted to prevent permanent hypoparathyroidism [15].

### Diagnosis of PC

Parathyroid cancer typically presents in individuals with an average age of 45–51 [16]. Approximately 90% of these cases are active endocrine tumors, while the remainder are non-functional [17], often exhibiting symptoms of compression or invasion of adjacent structures. Preoperative diagnosis of PC is challenging, as its manifestations frequently overlap with those of parathyroid adenoma (PA) or other parathyroid-related hereditary syndromes, such as multiple endocrine tumor syndrome (MEN) or hyperparathyroidism-jaw tumors syndrome (HPT-JT). Fine-needle aspiration (FNA) is not recommended for suspicious PC cases due to the risk of tumor implantation and metastasis along the needle track. The diagnosis of PC should be considered under the following conditions [18]: (a) serum calcium > 14 mg/dL; (b) serum PTH level 10 times higher than normal; (c) rapid onset of acute symptoms; (d) detection of metastasis on radiographic examination.

Despite advances in various imaging techniques, such as 18-fluorocholine positron emission tomography/computed tomography (18 F-PET/CT), PET/magnetic resonance imaging (PET/MRI), and four-dimensional CT (4D-CT), the classic combination of 99mTc-MIBI single-photon emission computed tomography/computed tomography (SPECT/CT) and cervical ultrasonography (cUS) remains the first choice for imaging examinations [2]. In cUS, PC exhibits the following characteristics: relatively larger size (i.e., > 3 cm), inhomogeneous structure, thick capsule, lobulated or irregular borders, suspicious vascularity and calcification, and signs of local invasion [19–21]. However, these features may not always be classical or specific. Most parathyroid carcinomas are suspected intraoperatively and definitively diagnosed postoperatively, often without distinctive preoperative signs. In our case, the auxiliary imaging examination results were contradictory: CE-US identified a parathyroid adenoma in the middle and lower left thyroid lobe, while 99Tc-sestamibi SPECT localized the lesion to the posterior tubercle of the superior left lobe of the thyroid. Postoperative histopathology is considered the gold standard for diagnosis, but it is limited by the availability of postoperative pathological specimens. Therefore, it is crucial to maintain a high suspicion for PC.

Additionally, immunohistochemical biomarkers, especially the Ki67 index, can enhance the accuracy of diagnosing atypical parathyroid neoplasms [22]. Parafibromin, coded by the Cell Division Cycle 73 (CDC73) gene, induces cell cycle arrest by inhibiting cyclin D1. PTH expression is almost exclusive to parathyroid tissues rather than thyroid tissue, facilitating the differential diagnosis of the histological origin of the tumor. Moreover, positive expression of CgA and CD44, negative expression of TTF-1 and adenomatous polyposis coli (APC) protein, overexpression of cyclin D1, and reduced p27 protein expression all support a PC diagnosis [23].

It is also worth mentioning that intraoperative PTH (ioPTH) determination can assist surgeons in determining whether to accurately remove target parathyroid gland with abnormally high secretion during surgery. According to the Miami criterion or Vienna criterion, the significant reduction of ioPTH eliminates the need to continue searching for other highly functional tissues during intraoperative neck exploration, which is particularly recommended in multiglandular diseases [24, 25]. However, some experts consider ioPTH monitoring is not necessary, for the false-negative results, false-positive results, surgical efficiency, medical cost burden, etc [26]. What’s more, in a recent prospective study, probe-based near-infrared autofluorescence has been proved a valuable intraoperative auxiliary tool in parathyroid gland identification [27].

### Management of PC

Parathyroid carcinoma is a malignant tumor characterized by a high degree of invasion. Its pathological features include invasion of adjacent anatomical tissues, vascular and lymphatic systems, intraneural infiltration, and even distant metastasis. If malignant characteristics are observed during neck exploration, care should be taken to ensure the complete removal of suspicious lesions while preserving tumor encapsulation. The detection of PC often relies on intraoperative observation, and surgical decision-making significantly influences prognosis. In our case, for instance, the presence of dense adhesions in the right thyroid lobe during surgery raised suspicions, prompting the consideration of an invasive manifestation of a malignant tumor originating from the thyroid or parathyroid. The decision to perform en-bloc resection avoided the need for a second surgery. A brief summary of recent management strategies for PC coexisting with PTC is presented in Table 1.

**Table 1:** Features and surgical management of patients with parathyroid carcinoma and papillary thyroid carcinoma

Authors	Year	Age, Sex	Calcium (mg/dl)	PTH (pg/ml)	FNA	PC Suspicion preoperatively	Indication of surgery	Carcinoma location	Parathyroid Size (mm)	PTC location, size(mm)	Surgical Treatment	Outcome
Ibtissem Ben Nacef, et al. [37]	2022	60, female	13.83	569	No	No	PHPT, cervical swelling	upper pole of the left thyroid	40	2 PTMC in left lobe, unknown	total thyroidectomy, left parathyroidectomy, central and lateral cervical lymph node dissection	normal serum calcium, no recurrence (9-month follw-up)
Hannah Daniel, et al. [38]	2022	31, male	unknown, 13.5 after the first operation	171	Yes	No	PHPT, cervical mass	left inferior thyroid lobe	25	/	**1**.local excision, partial left thyroid lobectomy; **2.** total thyroidectomy, lymph node dissection VI + right II ∼ V + left II ∼ IV, excision of superior right and left parathyroid; **3.** neck exploration with left parathyroidectomy and left revision thyroidectomy	Pulmonary metastasis
Koki Otake, et al. [30]	2021	71, male	11.5	131	Yes	Yes	NG + PHPT	right thyroid lobe	15	/	Right thyroid lobectomy and central neck lymph node dissection	normal PTH, no recurrence (5-month follw-up)
Nadia De Falco, et al [39]	2021	63, male	13.3	164	No	No, PA?	MNG	posterior thyroidal left basal lobe	12	left lobe 8; right lobe 6	total thyroidectomy	normal serum calcium, PTH, no recurrence (7-year follw-up)
Luca Foppiani, et al [40]	2021	67, male	unknown	unknown	Yes	No	PTC, PHPT	left thyroid nodule	unknown	left thyroid lobe, unknown	total thyroidectomy	normal serum calcium after surgery
César Ernesto Lam-Chung, et al [41]	2020	50, female	14.3	1160	No	Yes	PHPT	left thyroid lobe	19	left thyroid lobe,13 (pT1bN0M0)	en bloc resection of the parathyroid mass and left thyroid lobe and central lymph node compartment dissection	normal serum calcium 6 weeks postoperatively
Natalie Poortmans, et al [42]	2019	26, female	13	267.5	Yes	No, PA?	hypercalciuma	lower pole of the left thyroid	28	/	total thyroidectomy	no recurrence (5-year follow up)
Ovie Edafe, et al [43]	2019	46, female	>12	>65	Yes	No	PHPT	Adhesive right parathyroid mass	33.4	right thyroid lobe,	**1.**resection of right parathyroid mass, right thyroid lobe, right central compartment tissue and right recurrent laryngeal nerve;**2.**completion thyroidectomy	no recurrence (12-year follow up)
Noran Alharbi, et al [44]	2018	63, male	16.68	168.2	Yes	No, neuroendocrine tumor?	PHPT neuroendocrine tumor?	left thyroid lobe	27	unknown, 0.5	total thyroidectomy with central and left neck node dissection	recurrence and metastasis several months postoperatively
Meera Balakrishnan, et al [45]	2017	60, female	12.5	1721	Yes	Yes	PHPT, cervical mass	involving both lobes of thyroid and abutting the circumferential margin	45	/	total thyroidectomy	PTH 190.3 pg/mL, serum calcium 11.9 mg/dL postoperatively
Kursat Dikmen, et al [46]	2017	57, male	11.4	118	No	No	pulmonary embolism ; mediastinal mass	mediastinal mass and left inferior parathyroid	30, 21	PTMC left thyroid lobe, unknown	**1.**excision mediastinal mass thoracoscopically; **2.**Left parathyroidectomy, left thyroid lobectomy, isthmectomy, unilateral central lymph node dissection	normal serum calcium, PTH postoperatively
Cho-Ok Baek, et al [47]	2017	68, female	12.8	1247	Yes	Yes	PHPT	left inferior parathyroid	42	left thyroid lobe, 5	left inferior parathyroidectomy gland and left hemithyroidectomy	normal serum calcium, PTH, (6-month follw-up)
Alfredo Campennì, et al [48]	2016	65, female	10.8	160	No	No	PHPT, autonomously functioning thyroid nodules	right inferior parathyroid	21	MNG, 12	total thyroidectomy and right inferior parathyroidectomy	normal serum calcium, PTH, no recurrence (1-years follw-up)
Leila Chaychi, et al [49]	2010	79, female	11.4	89	Yes	No	Multifocal papillary thyroid carcinoma	left thyroid lobe	50	right thyroid lobe, 24, 17	total thyroidectomy and left-sided parathyroidectomy	normal serum calcium (6-month follow-up)

Complete surgical resection is the gold standard for curing PC, which entails total tumor resection, ipsilateral thyroidectomy, and central neck dissection [28, 29]. If intraoperative observation reveals abnormal invasion behavior of suspicious nodules into surrounding tissue structures, the resection of the invaded thyroid and adherent tissue is necessary [30], regardless of whether the concern is a malignant thyroid tumor or a malignant parathyroid tumor. In cases of persistent postoperative symptoms, the possibility of PTH secretion by metastatic parathyroid carcinoma should be considered [18].

If postoperative pathological confirmation indicates that the initial surgical intervention was insufficient, timely additional surgery should be pursued to maximize the chance of healing [31]. The treatment of lymph nodes remains controversial; some advocate for the routine dissection of ipsilateral central lymph nodes as a preventive measure, while others argue that intervention is necessary only if lymph node involvement is identified [32].

Cases where patients refuse surgery or resection margins are positive during the initial operation, radiotherapy may serve as an alternative therapeutic approach. Radiotherapy is essential for reducing the risk of local recurrence [33] and increasing disease-free survival [34]. Other treatment options include the calcimimetic drug cinacalcet and bisphosphonates, which can benefit patients with hypercalcemia as first-line treatments. Denosumab may stabilize calcium levels in cases of refractory hypercalcemia caused by parathyroid carcinoma [35].

Long-term prognosis for PC patients who undergo surgery remains poorly documented. However, it is evident that en-bloc resection with cancer-free resection margins during the initial operation is crucial for ensuring an excellent survival rate. Ten-year overall survival is estimated at 60–70%, as the tumor typically exhibits relatively slow growth [32]. Despite the rarity of intrathyroidal PC or the suspicion of invasive biological behavior of malignant carcinoma during intraoperative observation, surgeons must remain vigilant for the possibility of intrathyroidal PC and carefully examine the specimen to ensure negative margins. In our case, the patient experienced a successful recovery without complications or discomfort during the six-month follow-up period. Prognosis-related research has shown that, compared to en-bloc resection, conservative local excision results in a 3.5-fold higher risk of positive margins and a 6.4-fold higher risk of in situ recurrence [36]. Patients with recurrence require reoperation, which presents challenges in identifying and separating tissues and an increased risk of complications due to postoperative adhesion or metastasis. When distant organ metastases occur, such as in the liver, radiofrequency ablation, resection of liver metastases, or segmental hepatectomy can significantly improve symptoms.

## Conclusion

In summary, we have presented a case involving PTMC, ectopic parathyroid carcinoma, and multiple nodular goiter in the right thyroid lobe, coexisting with a left-sided parathyroid adenoma. A literature review was conducted, emphasizing the challenging difficulties in the diagnostic workup and surgical management of PC. Preoperative examination results, to some extent, serve as a reference; however, surgeons must make flexible decisions based on intraoperative findings. Dense adhesions may suggest the presence of malignant thyroid tumors or parathyroid carcinoma, necessitating cautious handling to prevent incomplete resection, rapid recurrence, or secondary surgery following pathological diagnosis, which can impact patients’ quality of life.

The presence of ectopic parathyroid glands and synchronous PA adds to the complexity of preoperative diagnosis for PC. In cases with suspicious thyroid nodules or parathyroid glands, and markedly elevated parathyroid hormone and serum calcium levels, the coexistence of PC should be considered. Immunohistochemistry and molecular studies are advantageous for classifying parathyroid tumors. Because of the highly invasive tendency of PC, it is crucial to perform complete resection as early as possible to prevent metastasis to lymph nodes, adjacent structures, and distant organs.

## Data Availability

Data will be made available on request.
